# The Danish sports chiropractic landscape: an exploration of practice characteristics and salient developmental issues

**DOI:** 10.1186/s12998-021-00383-4

**Published:** 2021-06-29

**Authors:** Corrie Myburgh, Julie Andersen, Nicklas Bakkely, Jakob Hermannsen, Marcus Zuschlag, Philip Damgaard, Eleanor Boyle

**Affiliations:** Department of Sports Science and Clinical Biomechanics, University of Southern Denmark, Campusvej 55, 5230 Odense M, Denmark

**Keywords:** Chiropractic, Athletic injuries, Mixed methods, Multiple methods, Professional practice, Delivery of health care

## Abstract

**Background:**

As is the case around the globe, the Danish chiropractic community appears to be an active service provider for the athletic sub-population. However, a paucity of evidence elucidating the experiences, perceptions, and practices of individuals who identify as ‘sports chiropractors’ complicates strategic development efforts.

**Methods:**

A sequential mixed-methods study was conceptualized in which interview responses from seven purposefully selected stakeholders provided context and informed a national descriptive survey exploring practice characteristics and opinions regarding sports chiropractic among Danish chiropractors.

**Results:**

Thematic highlights included divided opinions on the criteria that define a sports chiropractor, the role of a chiropractor functioning beyond the clinic setting, and the need for a structured approach to developing sports chiropractic as a legitimate sub-specialty.

The survey response rate was 34.9% (227 of 651), with 27% of responders identifying as a ‘sports chiropractor’. Compared to non-sports chiropractors, sports chiropractors engaged in a significantly higher level of interprofessional practice (3.8 versus 2.7 partners), in particular medical doctors (*p* = 0.016) and personal trainers (*p* <  0.001). Whether participants identified as a sports chiropractor or not, there was consensus that a high-quality post-graduate qualification and continued education was important. Generally speaking, the framing of sports chiropractic into a protected title was not a priority.

**Conclusion:**

The Danish sports chiropractor tends to be male, has a specialist education and engages other chiropractors, medical practitioners and professional trainers more often as practice partners than generalist chiropractors. The position of the sports chiropractor as a ‘knowledgeable expert’ was seen as more important than establishing a protected title. Experiential training appears to be an untapped resource for developing real-world competency and gaining greater professional exposure. Given the potential for development across Europe, more focus is required on a strategic plan for embedding chiropractic professionals in inter-professional athletic health and performance practice settings.

**Supplementary Information:**

The online version contains supplementary material available at 10.1186/s12998-021-00383-4.

## Background

Recent evidence generated in the English premiere football (soccer) league and highlighting injury-related decrements in performance [1] has again cast the spotlight on athlete health services. With the risk of lost earnings for each club estimated at £45 million per season, initiatives aimed at optimizing strategies in key domains of practice such as injury prevention, rehabilitation and health maintenance are likely to intensify [2, 3]. And as a consequence, it is conceivable that occupational groups, not typically included in service delivery, but offering novel solutions, may capitalize on this state of flux.

The chiropractic profession has a well-established tradition of managing athlete health, catering to the needs of both weekend warriors and sporting icons alike [4–6]. At present, service provider claims are primarily grounded in the literature describing practice and utilization within elite-level organized sport such as professional soccer and baseball [6, 7], and sporting events such as the Olympics and World Games [4, 6–9]. However, the specific role and value of chiropractors are still questioned by mainstream service providers, and their routine inclusion in service provision is rare [10–12]. At present, the inclusion of chiropractors as service providers is contingent on a shifting group of factors, including high-profile athlete perceived needs and satisfaction, organizational policy, exemplary performance of individual chiropractors and the acceptance of subordinate provider status [10].

To strengthen its position, sports chiropractors must be perceived as athlete-focused health care professionals, with unique and valuable expert knowledge and the competency to bring these to bear in inter-professional team-oriented practice environments [13, 14]. Additionally, this narrative must be developed among individuals with the power to drive health care practice innovation, such as athletic health and performance programme directors, elite professional club managers and key sport-related policymakers.

Systematic knowledge detailing professional characteristics, educational practice and political strategy in sports chiropractic currently offer very limited support for those invested in developing the position of the sports chiropractor [15]. Current paucities in the literature include practitioner profile and service provision data [5, 16], educational competency, regulation and coordination [12], and published works documenting desirable outcomes attributable to chiropractors in inter-disciplinary service delivery [6]. One point of departure in addressing this issue is to explore and describe existing practice experiences of chiropractors who have dedicated substantial parts of their professional lives to the care of athletes. It stands to reason that from a generalizable baseline, strategically meaningful hypotheses informing a global strategy for solidifying the professional reputation of sports chiropractors are more likely to emerge.

In this investigation, we focused on the European context. Despite the presence of multiple established chiropractic communities and centres of educational excellence [17, 18], little is known regarding the professional practice characteristics, education standards, and political organization specific to sport-related practice. Our study aimed to explore sports chiropractic through the practice experiences and salient developmental issues relevant to the Danish chiropractic community.

## Methods

### Study context

The Danish chiropractic community consists of approximately 650 practitioners [17], and although among the most regulated in Europe, individuals are not obliged to register as specialists in athletic health care [18]. The number of practitioners with a special interest in sport-related practice is therefore unknown. A special interest group (SIG) for sports chiropractic engages practitioners in sport-related practice activities. No local organization claims authority as a standard-bearer for sport-related post-graduate education, although such education is available through both the Danish Sports medicine and Chiropractic associations [19].

### Study design

This study was conceptualized as a two-phased exploratory sequential mixed-methods study. This approach was deemed appropriate, as the relatively complicated phenomenon of interest (sports chiropractic) was under-investigated, requiring qualitative data to develop a greater understanding of relevant domains of practice. Furthermore, data derived from phase 1, informed the development of and contributed to constructing validity of the survey instrument (phase 2) [20, 21].

#### Phase 1

##### Aim

To explore the notion of sports chiropractic and understand issues relevant to its development.

##### Researcher team position

The research team viewed this phase of the study through a constructivist lens, seeking to co-create meaning out of participant reflections of their professional practice experiences [22]. As chiropractors, the investigators responsible for data collection (JH and NB) approached respondents from an insider perspective [23].

##### Participants and sampling

A purposive sample strategy was followed to identify information-rich cases relating to the phenomenon of interest [24]. This type of non-probability sampling is typical of studies exploring relatively unknown research areas, where investigators aim to develop knowledge in a limited timeframe. In this regard, the SIG for sports chiropractic mediated access to practitioners to shed light on the topics of interest. A general letter of invitation was e-mailed to potential participants who then opted into participating. Snowballing was used as a secondary strategy to solicit responses from individuals with relevant knowledge regarding issues concerning education and politics. We anticipated that a sample of approximately 10 responders (*n* = 10) would be required to develop the thick descriptions required during thematic analysis [25].

##### Data collection and management

JH and NB conducted audio-recorded semi-structured individual interviews, either in-person or via VOIP platform (Skype). An interview guide was devised, consisting of six open-ended questions designed to explore the central question of what it means to be a sports chiropractor in Denmark. We used existing literature and input from the local sports chiropractic association as inspiration to develop interview questions encouraging feedback on education, practice and politics [26]. The list was narrowed down at a consensus meeting, during which input was presented and discussed by the research team. Probes supported all questions.

Once completed, interview raw data were transcribed verbatim by JH and NB into word text documents and anonymized by removing all identifying descriptors. An identification code was assigned to each respondent to facilitate the analysis, for example, ‘CH1’. Only audio was recorded during the interviews, and these files were deleted after the transcripts were generated.

##### Data analysis

A thematic analysis was conducted based on the framework method [27]. Moreover, a constant comparison approach of applying merging codes across all interview data was followed [28]. Two investigators (JH and NB) independently coded two interviews. Their coding was then compared, and a composite codebook developed, with a third investigator (CM), acting as referee. The composite codebook was applied deductively to subsequent interviews. If new codes were adopted, these were applied retroactively to all interviews. Code families were created to organize the data further, and axial codes were assigned to text containing latent meaning to develop patterns across the data [28]. Finally, a visual network was created to map the key themes. Once JH and NB had presented their network to the rest of the investigative team, adaptations were made based on group consensus. Due to the relatively low number of interviews and the proximity of the research team, the analysis was not conducted with computer-assisted analysis (CAQDAS) software [29].

A member check was conducted with one participant to ensure that the respondent’s voice was recognized in the findings [30].

##### Trustworthiness

Our strategies to establish confidence in the process and analysis of data included soliciting ‘thick descriptions’, researcher and data triangulation, member checking and reflexivity [31].

#### Phase 2

##### Aim

To describe and explore practice characteristics and opinions of sports chiropractors across the Danish chiropractic community.

##### Sampling

A population-based, cross-sectional approach was followed by surveying all chiropractors registered with the Danish chiropractic association (DCA). As more than 90% of chiropractors are members of the DCA, this approach was deemed appropriate to generalizable describe perceptions and practice trends.

##### Survey instrument

We took inspiration from previous questionnaires and salient issues identified in phase 1 of the study when designing our survey instrument. Specifically, we developed items relating to demographic data, professional involvement in sports practice, competencies, and patient base characteristics from existing literature. We included items covering interprofessional practice, association with sports clubs/teams, and frequency and level of athlete typically treated based on the interviews. Moreover, salient issues identified during phase 1 were transformed into quantifiable opinion statements, in which respondents could indicate their degree of agreement/disagreement.

Once formulated, the instrument was piloted for face and content validity. Regarding the former, two individuals unrelated to the project and no knowledge of sports chiropractic completed the questionnaire, providing feedback on sentence structure and clarity. Regarding the latter, two chiropractor stakeholders with a particular interest in athletic healthcare completed the questionnaire, focusing on linguistic content. Once revised, the final questionnaire was approved by the Chiropractic Knowledge Hub for distribution (see also Additional file 1).

##### Data collection and management

Through NIKKB, the survey was converted to an electronic platform (SurveyExact) and distributed through a group e-mail. This approach ensured participation was anonymous as individuals could voluntarily opt-in. The first e-mail was sent out on the 12th of March 2020, with two reminders following four-day intervals.

##### Data analysis

The sample was divided into sports chiropractors (SC) or not (non-SC) based on answering ‘yes’ to the following question ‘Do you consider yourself a sports chiropractor?’. The results were presented as descriptive statistics with percentages and 95% confidence intervals or means and 95% confidence intervals for the entire sample and by SC status. Differences in the proportions by SC status were found using either Pearson’s Chi-square test or Fisher’s exact test. Differences in the mean number of interprofessional partners was assessed using an independent t-test. We also determined the characteristics associated with identifying as an SC by conducting a backwards stepping multivariable logistic regression model with a *p* value to remove at 0.10. The following characteristics were entered into the model: sex, age group, the interaction between sex and age group, years since graduating from chiropractic training, post-graduate education (‘yes’ versus ‘no’) and frequency of treating amateur, semi-professional and professional level athletes. The assumptions for logistic regression model were satisfied. The level of statistical significance was set at 5%. All statistical analysis was done using SAS Studio Release 3.8 (Enterprise Edition).

## Results

### Phase 1

The first call for participation mediated by the SIG yielded 6 participants. One participant was included based on snowballing resulting in a sample of 7 (*n* = 7). The profile of the interview sample is presented in Table 1.

**Table 1:** Sample profile of interview respondents

Participant	Age	Sex	Years as sports chiropractor	Role(s)
1	60	M	33	Practitioner/politician/educator
2	46	F	13	Practitioner/educator.
3	49	M	24	Practitioner
4	55	M	30, 25 in elite sport	Practitioner/educator
5	44	M	15	Practitioner
6	40	F	11	Practitioner
7	55	M	18	Practitioner

Interview data were captured during February 2020, 4 being in-person and 3 via Skype. Interview duration ranged between 45 and 60 min, corresponding to a total of 6 h of transcribed audio data. Coding saturation appeared to occur after 5 interviews.

#### Reflexivity

The mediating function of the SGI simplified access to participants, and individuals appeared comfortable in their expert position. However, generally speaking, participants had difficulty reflecting on questions about education. The study findings were validated during the member check, and no further additions were deemed necessary.

#### Codes and themes

**Fig. 1:**
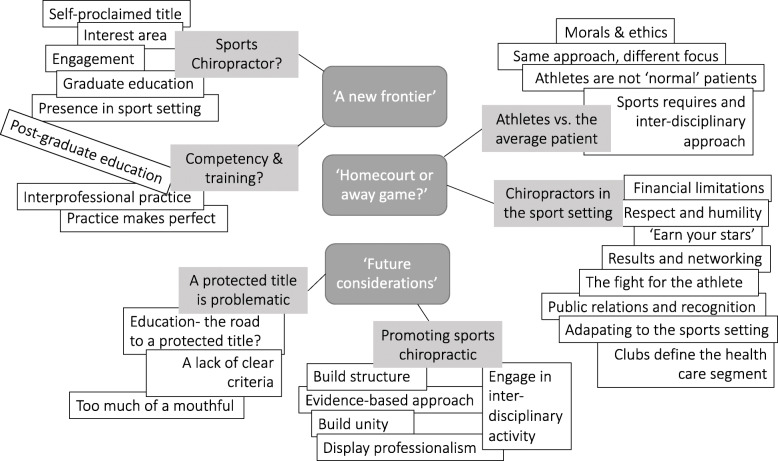
Thematic framework, illustrating central themes (dark grey), code families (light grey) and codes

As illustrated in Fig. 1, our analysis framework, constructed from 28 unique codes and 6 code families, centered around three themes: ‘A new frontier’, ‘Homecourt or away game?’ and ‘Future considerations’.

#### ‘A new frontier’

In the Danish context, the title of ‘sports chiropractor’ is currently a self-proclamation that carries none of the implicit criteria of a specialist practitioner. According to CH1:

… everyone can call themselves a sports chiropractor … it’s a personal title, the subject has no protected title...

No consensus attitude appeared to exist regarding a working definition, criteria and practice characteristics associated with the title and, furthermore, no minimum standards of practice are mandated. As such, and according to CH2 and CH5, a sports chiropractor is simply ‘[A chiropractor] that has cultivated a ‘special interest’ and has some level of ‘engagement’ in sports.

Juxtaposed perspectives were observed concerning a need for specialist knowledge, with CH1 arguing that:

So today everyone can call themselves a sports chiropractor … because they have received the basic training [as a chiropractor] and can handle treatment of musculoskeletal diseases and disorders.

In contrast, CH4 contended that part of the competencies required in dealing with athletes could only be acquired beyond the general clinical setting. Thus, for CH4:

I think there are some [unique] … skills and … knowledge one should have as a sports chiropractor. So yes, I think it’s an important … that you have to take some training.

Respondents also disagreed on whether the sports chiropractor, per definition, should practice in an external athletic environment and, furthermore, how much of their time should be dedicated to athletic health care. According to CH6:

Standing 37 h a week behind four white walls, does not make you a sports chiropractor. I believe that where you learn, ... where you get even sharper, … and get your fingers in it … [is] in the environments where there are athletes.

Working in a different setting made the interprofessional contact more likely, which according to CH4 and CH7, was integral to success in this area of practice. However, according to CH5, prioritizing attendance at workouts and matches is ‘traditionally the role of a physiotherapist,’ and that particular service provision should occur in the usual out-patient care mode.

At present, the notion of the sports chiropractor and what athletic practice in a Danish chiropractic context entails does not appear to be defined through title designation, specialist education, mode of practice or level interprofessional collaboration.

#### ‘Homecourt or away game?’

The nuanced management of athletes was associated with skillful communication and understanding the context in their experience rather than the clinical acumen. This view is exemplified by CH1, who stated:

… it doesn’t really look that much different … as I see it … assessing tissue overload or damage to the musculoskeletal system … it’s about meeting, the person who has the injury, in the place they are. There is a difference between whether it is a 65-year-old amateur who is training to run 10 km [and] the national team player in handball who comes in and says: “In 14 days I will go to the European Championships”.

The communication issue also extends into interprofessional practice, as the rapid feedback from team members facilitates agile management responses. According to CH5:’ … sometimes something more is needed, and it is probably more often with an athlete.’

For CH7, the world of sport ‘is an elite world’, and athletes tend to seek care from individuals with a reputation for getting results. Accordingly, to succeed, the sports chiropractor must go out and ‘earn your star on your shoulders … you do not get them because you come and say you are a chiropractor … ‘.

However glamorous being a ‘travelling’ sports chiropractor may sound, CH6 points out that financially speaking, this role cannot be sustained indefinitely and that this investment serves purely as a means of attracting athletic patients to the clinic.

It appears then that the importance of understanding the athlete, developing an interprofessional approach and creating a reputation is a process. Consequently, the sports chiropractor is likely to go through phases in their professional practice where they travel and are compelled to work outside of their regular clinical context.

#### ‘Future considerations’

The notion of a sports chiropractor emerging as a recognized specialist, perhaps with ‘special privileges’ emerged as a salient issue among participants. And strategies on how this may occur were not apparent from the data. However, three touchstones were observed. Specifically, from their responses, our participants encouraged development in practitioner participation, evidence-driven practice, and fostering a structured professional network of engaged practitioners.

Regarding participation, it was argued that the issue of a ‘protected title’ was a moot point as the priority in developing athletic health practice in the foreseeable future for chiropractors should be inclusion. In this regard, CH5 stated:

… the more people on that bandwagon the better it is right now. In the long run, it may well be that we need to have some criteria …

The consensus appeared to exist that, given the chiropractic profession’s successes in general clinical practice, a research-driven, evidence-based approach must be supported to promote and spread chiropractic services within the Danish athletic community. This view is illustrated by CH1, who stated:

… there are very few [specialists] who have a sports medicine Ph.D. … there is virtually no one with a focus on exactly this in sports … qualifying and formalizing one’s qualifications it’s just a stamp of approval. And it will just make things easier in the long run, in terms of gaining authority and respect.

Finally, to foster the development of a unified professional group, CH4 argued that it is crucial to create a practice structure and network that promotes the transfer of specialist knowledge from one generation of practitioners to the next. In this regard, CH4 offered the following discourse:

I think that what is important to gather [existing] sports chiropractors and make them realize that they are ambassadors for sports chiropractic, and how big a role and real duty [they] have, especially those ‘who sit a little high on straw and have joined clubs’, … you should get involved in the association and collaborate … how do we involve new people? How can I maybe help a young chiropractor interested in sport to maybe come in and observe me? Maybe get a job in a smaller club, and maybe work his way up...

Developmentally speaking, our respondents appeared to prioritize professional solidarity in the development of athlete-related service provision. In this regard, a strategy of claiming expert status by generating new formal knowledge was suggested due to the previously brokered successes for the profession. Additionally, transferring formal knowledge from one generation to the next through professional apprenticeship (internship) emerged as key to future development.

### Phase 2

**Fig. 2:**
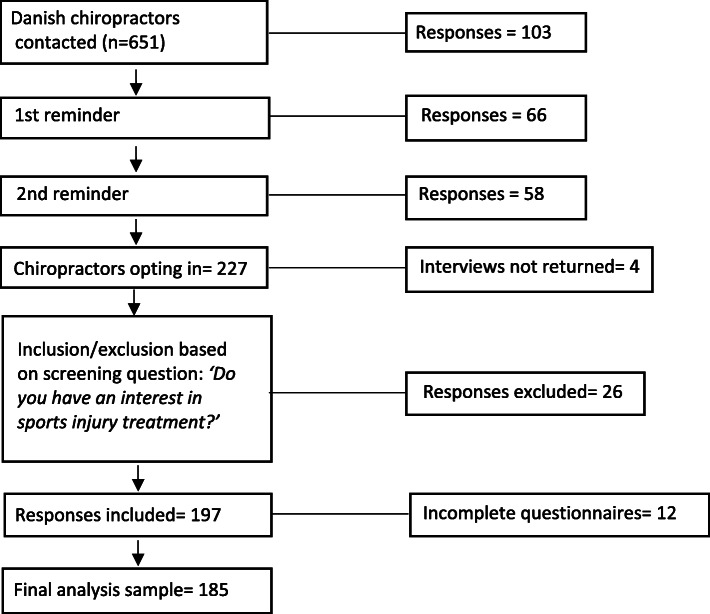
Flow chart of survey population sampling

Two hundred and twenty-seven of the 651 chiropractors registered with the DCA opted in for the survey for a response rate of 34.9% (see also Fig. 2). From the 185 complete data sets, 50 individuals (27%) identified as SC.

#### Demographics and practice characteristics

As indicated in Table 2, significantly more men identified as SC. Moreover, 78% of the SC sub-group indicated having undertaken a formal post-graduate education, a significantly higher percentage than the 36% observed among the non-SC.

**Table 2:** Demographic characteristics

	All (95% CI)	SC (95% CI)	Non-SC (95% CI)	*p* value
Sex				
Female	49.7 (42.5, 57.0)	28.0 (15.1, 40.9)	57.8 (49.3, 66.2)	< 0.001
Age group				0.217
21–30 years	24.3 (18.1, 30.6)	12.0 (2.7, 21.3)	28.9 (21.1, 36.6)	
31–40 years	16.8 (11.3, 22.2)	20.0 (8.5, 31.5)	15.6 (9.4, 21.7)	
41–50 years	24.9 (18.6, 31.2)	30.0 (16.8, 43.2)	23.0 (15.8, 30.1)	
51–60 years	26.5 (20.1, 32.9)	30.0 (16.8, 3.2)	25.2 (17.8, 32.6)	
> 60 years	7.6 (3.7, 11.4)	8.0 (0.2, 15.8)	7.4 (2.9, 11.9)	
Years since graduation				0.177
0–5 years	27.0 (20.6, 33.5)	16.0 (5.5, 26.5)	31.1 (23.2, 39.0)	
6–10 years	10.3 (5.9, 14.7)	16.0 (5.5, 26.5)	8.1 (3.5, 12.8)	
11–15 years	10.8 (6.3, 15.3)	14.0 (4.0, 24.0)	9.6 (4.6, 14.7)	
16–20 years	15.7 (10.4, 21.0)	14.0 (4.0, 24.0)	16.3 (10.0, 22.6)	
> 21 years	36.2 (29.2, 43.2)	40.0 (25.9, 54.1)	34.8 (26.7, 43.0)	
Post-graduate education	47.0 (39.8, 54.3)	78.0 (66.1, 89;9)	35.6 (27.4, 43.7)	< 0.001
Years treating sports injuries				
0–5 years	31.4 (24.6, 38.1)	20.0 (8.5, 31.5)	35.6 (27.4, 43.7)	0.102
6–10 years	9.7 (5.4, 14.0)	16.0 (5.5, 26.5)	7.4 (2.9, 11.9)	
11–15 years	13.0 (8.1, 17.9)	16.0 (5.5, 26.5)	11.9 (6.3, 17.4)	
16–20 years	16.2 (10.9, 21.6)	12.0 (2.7, 21.3)	17.8 (11.2, 24.3)	
> 21 years	29.7 (23.1, 36.4)	36.0 (22.2, 50.0)	27.4 (19.8, 35.0)	

*SC* Sports chiropractor, *Non-SC* Non-sports chiropractor, (*CI*) 95% confidence interval limits for percent

More than half of the SC subgroup (54%) reported having a formal relationship with a sports club or team, significantly more than the 10.4% in the non-SC group (Table 3). However, this association often did not include remuneration, where 55.6% of the SCs received payment. The SCs had, on average, significantly more interprofessional partners than the non-SCs (3.8 versus 2.7, respectively). SCs and non-SCs also differed significantly concerning the proportion of patients that sought care for sports-related problems, with 72% of SCs indicating that more than 10% percent of their patients were sports-related, compared to 22% of the non-SCs. Moreover, the SC sub-group was significantly more likely to treat professional or semi-professional athletes. Apart from the use of shockwave therapy, SCs and non-SCs did not differ significantly with respect to the number and type of modalities they considered relevant in managing athletic injuries.

**Table 3:** Selected practice characteristics

	All (95% CI)	SC (95% CI)	Non-SC (95% CI)	p value
*Formal association with a sports club or team*	22.2 (16.1, 28.2)	54.0 (39.7, 68.3)	10.4 (5.2, 15.6)	< 0.001
*Renumeration (yes)*	13.0 (8.1, 17.9)	30.0 (16.8, 43.2)	6.7 (2.4, 10.9)	< 0.001
*Physical presence at club*	8.1 (4.1, 12.1)	24.0 (11.7, 36.2)	2.2 (0.0, 4.7)	< 0.001
*Activities involving sports chiropractic*				
University lecturing	12.4 (7.6, 17.2)	14.0 (4.0, 24.0)	11.9 (6.3, 17.4)	0.694
Research	10.8 (6.3, 15.3)	12.0 (2.7, 21.3)	10.4 (5.2, 15.6)	0.751
Clinical supervision	20.5 (14.7, 26.4)	36.0 (22.2, 49.8)	14.8 (8.7, 20.9)	0.002
Voluntary work	25.4 (19.1, 31.7)	46.0 (31.7, 60.3)	17.8 (11.2, 24.3)	< 0.001
Sports associations	21.1 (15.1, 27.0)	44.0 (29.7, 58.3)	12.6 (6.9, 18.3)	< 0.001
None of the above	46.5 (39.2, 53.7)	18.0 (7.0, 29.0)	57.0 (48.6, 65.5)	< 0.001
*Average number of interprofessional partners*	3.0 (2.7, 3.2)	3.8 (3.3, 4.2)	2.7 (2.4, 2.9)	< 0.001
*Common interprofessional practice partners*				
Physiotherapist	85.4 (80.3, 90.5)	92.0 (84.2, 99.8)	83.0 (76.5, 89.4)	0.122
Chiropractor	53.5 (46.3, 60.8)	68.0 (54.6, 81.4)	48.1 (39.6, 56.7)	0.016
Massage therapist	53.0 (45.7, 60.2)	62.0 (48.1, 75.9)	49.6 (41.1, 58.2)	0.134
Medical doctor	47.0 (39.8, 54.3)	66.0 (52.4, 79.6)	40.0 (31.6, 48.4)	0.002
Personal trainer	21.1 (15.1, 27.0)	42.0 (27.8, 56.2)	13.3 (7.5, 19.1)	< 0.001
Acupuncturist	14.6 (9.5, 19.7)	20.0 (8.5, 31.5)	12.6 (6.9, 18.3)	0.205
None	7.0 (3.3, 10.7)	4.0 (0.0, 9.6)	8.1 (3.5, 12.8)	0.327
*> 10% Sports-related patient component*	35.7 (28.7, 42.6)	72.0 (59.1, 84.9)	22.2 (15.1, 29.3)	< 0.001
*Level of athlete treated*				
Professional	15.7 (10.4, 21.0)	38.0 (24.1, 51.9)	7.4 (2.9, 11.9)	< 0.001
Semi-professional	25.9 (19.6, 32.3)	58.0 (43.8, 72.2)	14.1 (8.1, 20.0)	< 0.001
Amateur	79.5 (73.6, 85.3)	92.0 (84.2, 99.8)	74.8 (67.4, 82.2)	0.010
*Average number of modalities considered to be relevant*	6.8 (6.5, 7.2)	6.9 (6.3, 7.6)	6.8 (6.4, 7.2)	0.678
*How important is the use of shockwave therapy in the treatment of sports injuries?*	59.5 (52.3, 66.6)	72.0 (59.1, 84.9)	54.8 (46.3, 63.3)	0.035

*SC* Sports chiropractor, *Non-SC* Non-sports chiropractor, (*CI*) 95% confidence interval limits around point estimate

Increased odds of identifying as an SC were observed for males, having a post-graduate education, and treating sports professional and/or semi-professional patients in their clinical practice (Table 4).

**Table 4:** Characteristics that are associated with identifying as a sports chiropractor

Variable	Odds ratio (95% CI)
Being male	4.274 (1.730–10.526)
Having a post-graduate education	9.174 (3.497–23.810)
Often or always treating professional patients	3.301 (1.042–10.461)
Often or always treating semi-professional patients	5.186 (1.973–13.631)

#### Opinions about sports chiropractic

As illustrated in Table 5, the respondent group maintained similar views for critical opinions and attitudes regarding sports chiropractic. Specifically, less than half of the responder group appeared to consider ‘being physical’ present at training sessions or matches essential (34% (SC) and 42% (Non-SC), respectively). Moreover, the importance of high-quality post-graduate and continuing education was similar for both groups (80% (SC) and 82% (Non-SC), respectively). Finally, the notion of ‘a protected title of sports chiropractor’ was not highly prioritized among the respondents, with 40% of sports SC and 38% of non-SC considering this issue to be important or very important.

**Table 5:** Opinions and attitudes regarding sports chiropractic

	All (95% CI)	SC (95% CI)	Non-SC (95% CI)	*p* value
“It is important to be physically present in the club in connection with training when you have a chiropractic collaboration with a sports club and / or team”	39.5 (32.4, 46.6)	34.0 (20.4, 47.6)	41.5 (33.1, 49.9)	0.355
“It is important to be physically present in the club in connection with matches, shows, competitions, rallies, etc., when you have a chiropractic collaboration with a sports club or team.”	41.6 (34.5, 48.8)	36.0 (22.2, 49.8)	43.7 (35.2, 52.2)	0.345
“It is a good idea to have a high-quality continuing education in sports chiropractic”	81.6 (76.0, 87.3)	80.0 (68.5, 91.5)	82.2 (75.7, 88.8)	0.729
“It is a good idea to make the title ‘sports chiropractor’ a protected title.”	38.4 (31.3, 45.5)	40.0 (25.9, 54.1)	37.8 (29.5, 46.1)	0.783

*SC* Sports chiropractor, *Non-SC* Non-sports chiropractor, (*CI*) 95% confidence interval limits for percent

## Discussion

### Main findings

Based on our observations, 7.6% of the Danish chiropractic community maintains a special interest in sport-related practice. Being male, having a post-graduate education and treating professional and semi-professional athletes are characteristics currently associated with identifying as a sports chiropractor. Compared to non-sports chiropractors, sports chiropractors are significantly more involved with clinical supervision, voluntary work and sports associations. Moreover, sports chiropractors do not employ a broader range of treatment modalities compared to non-sports chiropractors, although the sports chiropractor group makes more frequent use of shockwave therapy. Finally, sports chiropractors engage other chiropractors, medical doctors and personal trainers significantly more often as professional practice partners.

Danish chiropractors do not consider a protected title for sports chiropractic as a current developmental priority. Instead, their focus appears to lie with claiming expert status through post-graduate education and research and increasing the level of involvement in sport-related practice across the profession.

Expert practitioners stressed the importance of professional apprenticeship experiences as key to penetrating the world of sport. However, this view was not mirrored in the general opinion with physical presence at training and competitive events not being considered particularly important.

### Profile and practice trends

When comparing our findings with available data [5, 16], the following practitioner profile and practice trends can be discerned:

A sports chiropractor is more likely to be male, to have a post-graduate education and to associate with sports clubs or teams where they are often active in ancillary voluntary work. Moreover, interprofessional collaboration [5] features prominently with collaboration including at least one sport and fitness industry partner. Frequent use of electro-modalities suggests the targeting of tissue repair as a more commonly occurring management goal [16].

### Legislated specialist versus knowledgeable expert

Excluding others from a particular area of practice, through, for example, a legally protected title, is a strategy long associated with the provision of expert labour and is commonly referred to as professional boundary work [32]. Notwithstanding its undoubtedly historical success, boundary work has come under increased criticism as anti-competitive behaviour undermining the ethos of patient-centered care [33]. Writing in the Australian context, Pollard et al. essentially endorsed this strategy, lamented the absence of a protected title for legitimately trained sports chiropractors, and arguing that excluding non-specialized chiropractors protects the best interest of patients [12]. Our data, however, provided a cautionary tale, pointing out the risk of intra-professional segregation as undesirable for the development of an immature area of specialization.

The Danish chiropractic community has successfully used formal knowledge production (research) to lever professional legitimacy [18]. Therefore, it is not surprising that study participants highlighted a ‘legitimacy through knowledge’ approach as an alternative means for claiming an expert position in sport-related practice. Our data suggested that a relative paucity of research and university-related activity currently exists among sports chiropractors. There was a need to be perceived for more expertise, particularly at the doctoral level. In this regard, our findings mirror existing literature, which strongly supports advanced study as one of the key pillars supporting specialization [5, 12, 16]. Additionally, our investigation underscored the importance of empirical research performed by chiropractors as key to supporting their claim as athletic health subject experts.

### Experiential learning

Finally, in organized sport, the value of professional internships as a mechanism to enhance employability is undeniable [34]. This form of experiential learning exposes young professionals to a live-work setting, allowing them to hone their skills and competencies. Additionally, potential employers have a risk-free opportunity to evaluate enthusiastic young candidates for potential employment within the organization. Our respondents alluded to this issue, observing that specialist status is not determined by formal education alone, but also resides in experiences derived from learning in the real world. To our knowledge, the professional internship as it pertains to sports chiropractic remains entirely underexplored in the formal literature.

### Strengths and limitations

Our study was conducted at the start of the first COVID-19 lockdown period (March 2020), and as such, our response rate may have suffered. However, despite this issue, we still solicited responses from more than a third of Danish chiropractors about a niche area of practice.

This investigation is the first to be conducted in the Danish context and uses quantitative and qualitative data to triangulate its findings. Therefore, the findings are more likely to be trustworthy, reflecting both the views of specialist stakeholders and general perspectives. However, although we achieved code saturation, the thickness of descriptions around post-graduate education was limited, and we could not explore this issue further due to the timeframe of the investigation. As such, the specificity of questionnaire items relating to this domain could have been improved.

We could have generated more questionnaire items based on the interview data, which may have shifted the focus of our findings somewhat. However, we were mindful of limiting the length of the questionnaire and striking a balance between the local context and comparing our findings with existing descriptive questionnaires.

### Future research

To build the case for awareness and inclusion of the sports chiropractor and to help capture the gage of industry leaders, we encourage formal initiatives that:

- Increase the involvement of female practitioners
- Explore other chiropractic communities across Europe; in particular ones describing practice characteristics and highlightinh developmental issues.
- capture the unique contributions of the chiropractor in inter-professional practice environments and
- develop advanced study in university settings that generate unique, empirical knowledge and provide experiential learning through professional internships

## Conclusion

Sports chiropractic represents an area of interest for approximately 8% of the current Danish chiropractic community. Profile wise, sports chiropractors tend to be male, report having specialist education and engage in significantly higher levels of interprofessional practice than their generalist counterparts. The position of the sports chiropractor as a ‘knowledgeable expert’ was seen as more important than establishing a protected title. Experiential training appears to be an untapped resource for developing real-world competency and gaining greater professional exposure. Given the potential for development across Europe, more focus is required on a strategic plan for embedding chiropractic professionals in inter-professional athletic health and performance practice settings.

## Supplementary Information

**Additional file 1.**

## Data Availability

Interview transcripts are available on request.
