# Supplementary material for: The Danish sports chiropractic landscape: an exploration of practice characteristics and salient developmental issues
Source: Chiropr Man Therap. 2021 Jun 29;29:22. doi: 10.1186/s12998-021-00383-4 (PMC8240076; doi:10.1186/s12998-021-00383-4)
Supplement: Supplementary file 1 — Additional file 1. [file 12998_2021_383_MOESM1_ESM.docx]

# **Appendix I**

**Interview guide**

**(Probing questions indicated with bullets)**

**Practice:**

1. What does it mean to be a sports chiropractor in Denmark?

- What characterizes the sports chiropractic approach to handling and treating athletes?
- How much of one's working hours should be related to sports-related injuries in a sports chiropractic context?

1. What are your thoughts on interprofessional collaboration in the treatment of athletes?

- What is the role of the sports chiropractor in interdisciplinary collaboration around the treatment of sports injuries?

1. How important is a formal collaboration with a sports club or organization for your work as a sports chiropractor?

- What are the advantages/disadvantages of affiliation with a sports club as a sports chiropractor?
- What is the level of commitment between you and the club/organization?

**Education:**

1. How important is postgraduate education for being sports chiropractor?

- At what level should the education lie?

**Politics:**

1. What can we do to increase the sports chiropractor's role in the Danish world of sports?

- Should the term "sports chiropractor" as a protected title?

**Other:**

1. What are the most important criteria for calling oneself a sports chiropractor?

# **Questionnaire (English translation)**

**Information about the project and consent:**

The aim of the study is to create a survey of sport chiropractic in Denmark.

Before you give consent to participate in the project, you must know what it entails:

- Participation is voluntary and you can withdraw from the project at any time
- No sensitive personal data is collected, only demographic data, such as age, gender and year of graduation.
- All information you provide to the project is treated confidentially. The information is only passed on to researchers and their students.
- Prior to publication, all information is anonymised so your contribution to the project does not reveal your identity.
- You can request access to your answers at any time and have a copy handed out. Furthermore, you can object to the processing of your data or make a complaint to the Data Protection Agency of the data processing.
- The information will only be stored in personally identifiable form for as long as it is necessary for the purposes of our research. They are then anonymized or deleted.

This statement of consent is the legal basis for us to use your information for research.

Best Regards

…..

I hereby give my consent to participate in the study

- Yes
- No

Do you have an interest in sports injury treatment within chiropractic?

- Yes
- No
- I don’t know

Is treatment / diagnosing of sports injuries part of your practice?

- Yes
- No
- I don’t know

**The clinic**

1. Which of the following practitioners have you had a professional referral relationship with related to sport injury treatment within the past 6 months?
   - Physiotherapist
   - Chiropractor
   - Masseuse
   - Acupuncturist
   - Osteopath
   - Craniosacral therapist
   - Psychologist
   - Doctor
   - Body SDS
   - Personal trainer
   - Other
   - None of the above
2. Are you as a chiropractor affiliated with a sports club and / or a team as a therapist?

- Yes
- No
- I don’t know

2a. Are you being paid for one or more of your chiropractic affiliations with sports clubs and / or teams?

- Yes
- No
- I don’t know

2b. Is physical presence in the club part of one or more of your chiropractic affiliations with sports clubs and / or teams?

- Yes
- No
- I don’t know

1. How much do you agree with the following statement: “it is important to be physically present in the club during training, when you have a chiropractic affiliation with a sports club or a team”?
   - Strongly disagree Collapsed strongly disagree, disagree and
   - Disagree neither into ‘Disagree’
   - Neither
   - Agree Collapsed agree and strongly agree into
   - Strongly agree ‘Agree’
2. How much do you agree with the following statement: “it is important to be physically present in the club during matches, shows, competitions, rallies etc., when you have a chiropractic affiliation with a sports club or a team”?
   - Strongly disagree Collapsed strongly disagree, disagree and
   - Disagree neither into ‘Disagree’
   - Neither
   - Agree Collapsed agree and strongly agree into
   - Strongly agree ‘Agree’

**Education:**

*The following questions is about your primary education and any continuing education / postgraduate courses.*

1. Have you graduated one or more of the following continuing education and postgraduate courses?
   - FICS (International federation of sports chiropractic)
   - IKE (Sports chiropractic continuing education from NIKKB)
   - Master
   - PhD
   - Other (Describe in textbox)
   - None
2. How much do you agree with the following statement: “It is a good idea to have a high-quality continuing education in sports chiropractic”?
   - Strongly disagree Collapsed strongly disagree, disagree and
   - Disagree neither into ‘Disagree’
   - Neither
   - Agree Collapsed agree and strongly agree into
   - Strongly agree ‘Agree’
3. How much do you agree with the following statement: “it is a good idea to make the title ‘sports chiropractor’ a protected title”?
   - Strongly disagree Collapsed strongly disagree, disagree and
   - Disagree neither into ‘Disagree’
   - Neither
   - Agree Collapsed agree and strongly agree into
   - Strongly agree ‘Agree’
4. Which of the following have you been involved in during the past 12 months? (Select all that apply)
   - University teaching
   - Research
   - Clinical supervision
   - Volunteer work
   - Sports organization or federation activity
   - None of the above
5. How much do you agree with the following statement: “it is a good idea to maintain your competencies and stay up to date in research related to sports injuries, if you as a chiropractor handle these”?
   - Strongly disagree Collapsed strongly disagree, disagree and
   - Disagree neither into ‘Disagree’
   - Neither
   - Agree Collapsed agree and strongly agree into
   - Strongly agree ‘Agree’

**Personal practice**

*The following questions is about your personal activities and clientele as a chiropractor*

1. How big an amount of your clientele comes with sports-related injuries on average weekly
   - 0%
   - 1-10%
   - 11-20%
   - 21-30%
   - 31-40%
   - 41-50%
   - >50%
2. How relevant do you think each of the following modalities is when treating sports injuries?
   - Extremity manipulation (very irrelevant, irrelevant, neither, relevant, very relevant)
   - Needling (very irrelevant, irrelevant, neither, relevant, very relevant)
   - Stretching modalities: MET / PNF stretch / active stretch (very irrelevant, irrelevant, neither, relevant, very relevant)
   - Other uninstrumented soft tissue treatment: trigger point treatment, massage, cross-fiber massage etc. (very irrelevant, irrelevant, neither, relevant, very relevant)
   - In-line traction (very irrelevant, irrelevant, neither, relevant, very relevant)
   - Ultrasound treatment (very irrelevant, irrelevant, neither, relevant, very relevant)
   - Shockwave treatment (very irrelevant, irrelevant, neither, relevant, very relevant)
   - Laser (very irrelevant, irrelevant, neither, relevant, very relevant)
   - Cryo therapy (very irrelevant, irrelevant, neither, relevant, very relevant)
   - Functional taping (very irrelevant, irrelevant, neither, relevant, very relevant)
   - IASTM / instrument assisted soft tissue mobilization (very irrelevant, irrelevant, neither, relevant, very relevant)

Collapsed very irrelevant, irrelevant, neither into ’irrelevant’. Collapsed relevant, very relevant into ‘relevant’

1. State the relevance of guidance in each of the following when dealing with patients with sports injuries
   - Medication (very irrelevant, irrelevant, neither, relevant, very relevant)
   - Diet / nutrition (very irrelevant, irrelevant, neither, relevant, very relevant)
   - Physical activity /fitness (very irrelevant, irrelevant, neither, relevant, very relevant)
   - Pain Counselling (very irrelevant, irrelevant, neither, relevant, very relevant)
   - Sports psychology (very irrelevant, irrelevant, neither, relevant, very relevant)
   - Social medicine (very irrelevant, irrelevant, neither, relevant, very relevant)

Collapsed very irrelevant, irrelevant, neither into ’irrelevant’. Collapsed relevant, very relevant into ‘relevant’

1. How often do you treat sportspeople or athletes of following levels in your practice?
   - Professional / elite (Never, rarely, sometimes, often, always, I don’t know)
   - Semi-professional (Never, rarely, sometimes, often, always, I don’t know)
   - Amateur (Never, rarely, sometimes, often, always, I don’t know)

Collapsed ‘always and ‘often’ together. Collapsed ‘never, ‘rarely’, ‘sometimes’ and ‘I don’t know’ together.

**Demographic data**

1. Your age?
   - 21-30 years
   - 31-40 years
   - 41-50 years
   - 51-60 years
   - 61-70 years
   - >70 years
2. Your gender?
   - Female
   - Male
   - Other
3. Years since graduation?
   - 0-5 years
   - 6-10 years
   - 11-15 years
   - 16-20 years
   - > 21 years
4. How many years of experience do you have with treating and / or diagnosing sports injuries?
   - 0-5 years
   - 6-10 years
   - 11-15 years
   - 16-20 years
   - > 21 years
5. Do you consider yourself a sports chiropractor?
   - Yes
   - No
   - I don’t know
